# Mobile Health Applications for Depression in China: A Systematic Review

**DOI:** 10.7759/cureus.27299

**Published:** 2022-07-26

**Authors:** Leping Huang, Victor W Li, Tao Yang, Jing Liu, Jill Murphy, Erin E Michalak, Zuowei Wang, Chee Ng, Lakshmi Yatham, Jun Chen, Raymond W Lam

**Affiliations:** 1 Psychiatry, Hongkou Mental Health Centre, Shanghai, CHN; 2 Department of Psychiatry, University of British Columbia, Faculty of Medicine, Vancouver, CAN; 3 Shanghai Mental Health Centre, Shanghai Jiao Tong University School of Medicine, Shanghai, CHN; 4 Psychiatry, University of British Columbia, Vancouver, CAN; 5 Department of Psychiatry, University of Melbourne, Melbourne, AUS; 6 Psychiatry, Shanghai Mental Health Center, Shanghai Jiao Tong University School of Medicine, Shanghai, CHN

**Keywords:** systematic review, wechat, china, depression, smartphones, mhealth, mobile health

## Abstract

Mobile health (mHealth) applications (apps) have the potential to increase access to mental health care. In China, there is growing interest in mHealth apps for depression. Our objective was to systematically review research on mHealth for depression in China to identify benefits and challenges.

A systematic literature search was conducted using Chinese and English databases in accordance with Preferred Reporting Items for Systematic Reviews and Meta-Analyses (PRISMA) guidelines. Randomized and nonrandomized clinical studies on mHealth apps and depression in China were included. Study quality was assessed using the Cochrane Risk of Bias tool.

Seven studies met the inclusion criteria with three randomized trials, two quasi-randomized trials, one clinical trial with an uncertain grouping method, and one study with a single-group design. All studies used the WeChat platform and included activities such as psychoeducation, self-management, supervised group chats, and/or remote contact with a healthcare team, in comparison to usual care. All studies reported significant and large benefits for outcomes, but the risk of bias was high.

There are few rigorous evaluations of mHealth apps for depression in China, with all included studies involving WeChat programs and most using WeChat to extend nursing discharge care for inpatients with depression. While these studies showed significant improvement in health outcomes as compared to usual care, the results remain inconclusive because of the high risk of bias. mHealth holds promise for increasing access to mental health care in China, but issues such as efficacy, scalability, patient and clinician acceptability, and data privacy must be addressed.

## Introduction and background

Major depressive disorder (MDD) is a common mental disorder affecting more than 280 million people worldwide, representing about 4% of the population [1]. In China, over 56 million people are estimated to suffer from MDD [2-3]. Depression was estimated to result in the loss of 5.8 million disability-adjusted life years (DALYs), an increase of 36.5% from 1990 to 2017, ranking it the 10th leading medical cause of DALYs in China [2].

Many efforts have been made to improve depression treatment in the past decades. As computer and Internet technology matured, physicians started to apply electronic health (eHealth) to mental health services, and eHealth practices are proving to be successful and cost-effective [4]. Recently, mobile phone use has dramatically increased and smartphones have become ubiquitous. In 2020, there were over 6 billion smartphone users globally [5]. Mobile health (mHealth) is defined as the practice of medicine supported by applications, or apps, for mobile devices such as smartphones and tablets [6]. mHealth differs from the broader term, eHealth (which includes telemedicine and internet-based resources), by incorporating mobile device capabilities such as multimedia technologies and on-device notifications. Hence, mHealth is more than simply a web browser or video conferencing program that is launched from a mobile device. mHealth services and apps for mental illness have shown evidence of effectiveness in many countries [7-9].

In China, mobile use has also skyrocketed. In 2021, China had over 1.03 billion internet users and 1.64 billion mobile phone subscriptions [10]; almost all internet users in China now go online using mobile devices. A recent Chinese survey on e-Mental Health found that 83% (n=133/184) of patients and family members, 92% (n=208/225) of mental health professionals, and 92% (n=162/177) of respondents from the general population reported high or relatively frequent use of mobile devices [11]. WeChat, the widely popular social media platform with over 900 million users [12], is embedded into the lives of most Chinese people, from booking appointments to paying for services and products. However, the application of mHealth interventions in China, especially in mental health service delivery, is nascent but seeing rapid growth. In 2016, a systematic review found 234 Chinese mHealth apps, but only three apps were used by psychiatrists [13]. By 2020, there were 843 apps about psychology, and 121,560 apps on psychological counseling available on online app stores [11]. Thus, there are considerable opportunities for mHealth in China.

Existing systematic reviews on mHealth focus on Western countries, Asian regions other than China, or on patients with other diseases [7,14]. Very few systematic reviews are relevant to mHealth for mental health in China, and none have focused specifically on depression. To address this knowledge gap, we conducted a systematic review of mHealth interventions for depression in China, with an aim to identify the benefits of mHealth and opportunities and challenges for future research.

## Review

Methods

Search Method

We used methods consistent with the Preferred Reporting Items for Systematic Reviews and Meta-Analyses (PRISMA) guidelines and checklist [15], but the protocol was not registered. A systematic literature search was done using combinations of the keywords “CHINA + Mental Health / Depression / Online (Intervention) / eHealth / Internet (based) / Web-based / Mobile Health / Mobile App / mHealth / WeChat / iPad / iPhone”, both in English and Chinese. The search was conducted in three popular Chinese databases: www.cnki.net, www.cqvip.com, and www.wanfangdata.com.cn/index.html, and two English databases: PubMed and Google Scholar, from inception to December 31, 2020. We did backward reference chaining by searching through bibliographies of relevant articles.

Selection Criteria

We included randomized or non-randomized studies based on the criteria in Table 1. Study selection was conducted independently by two reviewers; disagreements were resolved by consensus with a third reviewer.

**Table 1:** Inclusion and exclusion criteria for studies

Category	Criteria
Inclusion:	(1) Involved an mHealth app or intervention
	(2) Targeted major depressive disorder or depressive symptoms
	(3) Conducted in mainland China or had data on users in China
Exclusion:	(1) Involved other illnesses that were primary (e.g., epilepsy with depressive symptoms)
	(2) Only used the phone feature (e.g., telephone-delivered psychotherapy)
	(3) Included only populations in Hong Kong or Chinese immigrants in other countries (because of differences in health care systems)
	(4) Involved only online applications (e.g., internet-delivered psychotherapy), even though these can be viewed on a smartphone browser, our focus was on mHealth apps

Assessment of Risk of Bias

We summarized the risk of bias of individual randomized studies using the Cochrane Risk of Bias tool [16]. The risk of bias domains assessed was random sequence generalization, allocation concealment, blinding of participants and study personnel, blinding of outcome assessment, selective reporting, and other bias. The assessment was conducted independently by two assessors with disagreements resolved by consensus with a third assessor.

Results

Screening and Selection

**Figure 1:**
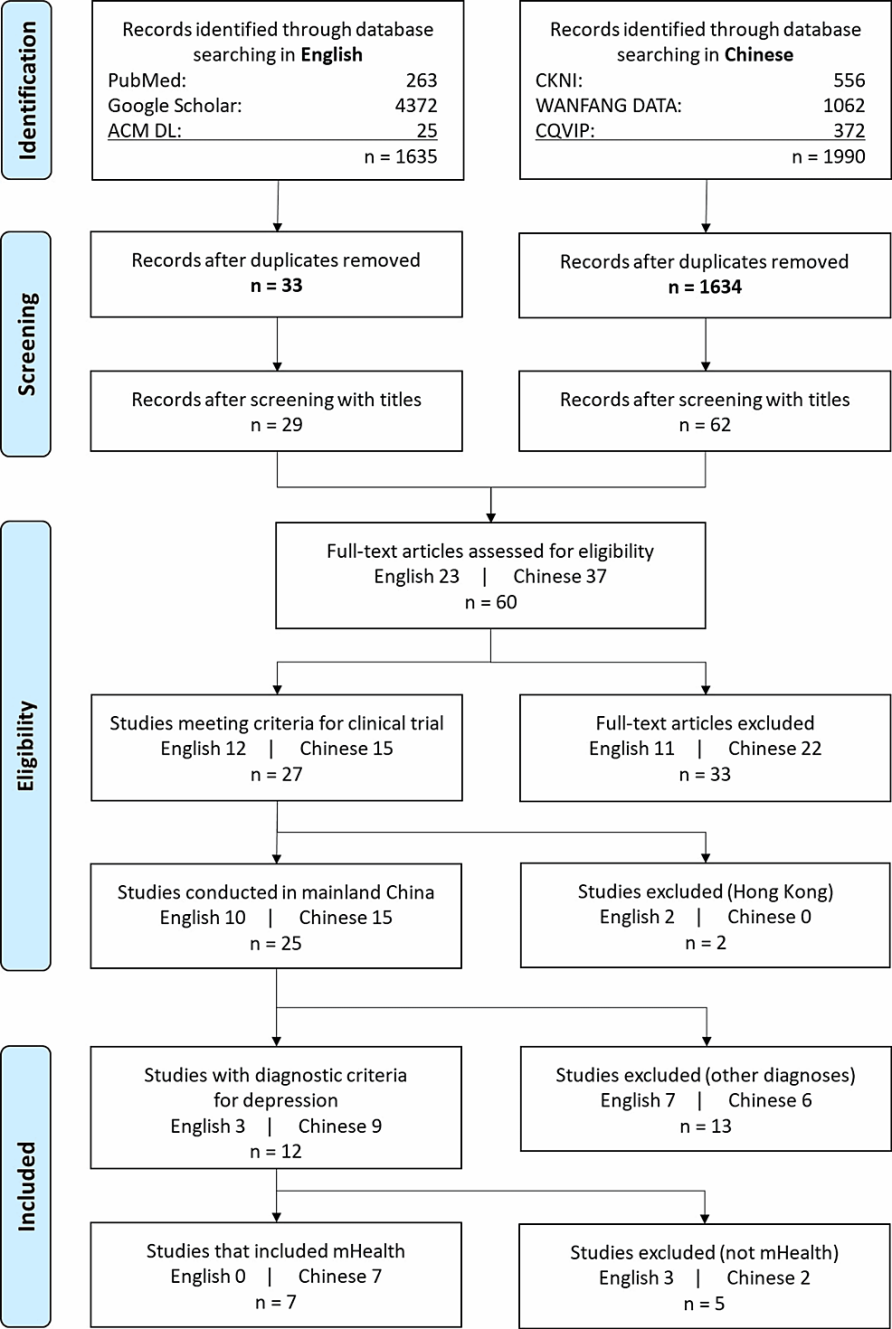
PRISMA flow diagram ACM DL, Association for Computing Machinery Digital Library; CKNI, China National Knowledge Infrastructure; CQVIP, Chongqing VIP Information database; PRISMA, Preferred Reporting Items for Systematic Reviews and Meta-Analyses

Figure 1 shows the PRISMA flow diagram. After screening for inclusion/exclusion criteria and removing duplicates, we found 12 papers (9 written in Chinese and 3 in English) that appeared suitable for inclusion. Upon detailed review, we excluded one study of a telephone-only intervention and four papers on internet-only applications, such as online discussion groups and online data reporting, leaving seven eligible papers addressing mHealth for depression in China.

Study Characteristics

Table 2 summarizes the key features of the included studies. All seven included studies used the WeChat platform and were published in Chinese journals. There were three randomized controlled trials (RCTs), two quasi-randomized trials, one clinical trial with an uncertain grouping method, and one study with a single-group design. The period of research spanned from 2014 to 2019 and research sites covered the coastal and inland provinces of China, including the cities of Qingdao (Shandong province), Zhenjiang (Jiangsu), Guangzhou (Guangdong), and Luohe (Henan). The included studies were conducted primarily in psychiatric hospitals (which are named mental health centers in China) and general hospitals. For diagnostic criteria, four studies used the International Classification of Diseases (ICD-10), two studies used the Chinese Classification and Diagnostic Criteria for Mental Disorders, 3rd edition (CCMD-3), which has diagnostic categories and criteria similar to the ICD-10, and one study did not report the diagnostic criteria. The participants in the seven studies were primarily hospitalized patients who had been recently discharged; outpatients were included in only one study. The sample size of individual studies ranged from 60 to 212. Outcome assessments included various clinician-administered symptom scales and patient-rated scales. Because of the considerable heterogeneity in patient populations, study methods, and outcomes, quantitative synthesis was not possible. Instead, the studies are summarized in a narrative review.

**Table 2:** Characteristics of included studies ADL, Activities of Daily Living scale; BMQ, Beliefs about Medical Questionnaire; CCMD-3, Chinese Classification and Diagnostic Criteria for Mental Disorders, 3rd edition; GQOLI-74, Generic Quality of Life Inventory-74; GSES, General Self-Efficacy Scale; HAM-A, Hamilton Anxiety Rating Scale; HAM-D, Hamilton Depression Rating Scale; ICD-10, International Classification of Diseases; ITAQ, Insight and Treatment Attitude Questionnaire; MMAS; Morisky Medication Adherence Scale; RCT, randomized controlled trial; SAS, Self-rating Anxiety Scale; SDS, Self-rating Depression Scale; WHO-QOL, World Health Organization Quality of Life scale

Author, year	City, Province	Design	Participants	Diagnostic criteria	Inclusion criteria	Enrolled Number	Gender n, M/F	Mean age years (SD)	Illness duration (SD)	Intervention Condition (duration)	Comparison Condition	Outcome Assessments
Ai X et al 2019 [17]	Jinan, Shandong Province	Quasi-randomized Trial (patients grouped by order of discharge)	Patients with depression discharged from hospital	ICD-10	Diagnosis of depression by ICD-10	79 intervention group; 77 comparison group	Intervention group 37/42; Comparison group 34/43	Intervention group 32.80 (9.40); Comparison group 33.20 (9.80)	Intervention group 6 months to 10 years; Comparison group 6 months to 5 years	WeChat program on psycho-education, self-management group chats with problem-solving, social skills and vocational coaching, medication guidance, supervised by nurses (1 year)	Discharge nursing as usual	SDS, SAS, Medication adherence
Huang C et al, 2018 [18]	Guangzhou, Guangdong Province	RCT	Patients with depression and anxiety discharged from the hospital	CCMD-3	SDS >53; SAS >50; before treatment in hospital	50 per group; 2 groups	Intervention group 28/22; Comparison group 31/19	Intervention group 37.7 (11.5); Comparison group 36.8 (11.0)	Intervention group 21.4 (5.7) months; Comparison group 21.7 (5.8) months	WeChat program for patients and families on psycho-education, self-management, medication adherence, and group chats supervised by nurses (6 months)	Discharge nursing as usual	SDS, SAS, WHOQOL-100; Study-specific medication adherence scale.
Lin Z et al, 2019 [19]	Liaocheng, Shandong Province	RCT	Patients with depression in remission discharged from the hospital	ICD-10	18-60, in remission; HAM-A <18; Junior high reading level	45 per group; 2 groups	Intervention group 16/19; Comparison group 17/22	Intervention group 36.99 (2.3); Comparison group 37.24 (2.4)	Intervention group 4.17 (0.7) years; Comparison group 4.21 (0.6) years	WeChat program for patients and family on psycho-education, group activities and multimedia, medication management, and group chats supervised by nurses (6 months)	Discharge nursing as usual	17-item HAM-D, HAM-A, ADL, and relapse and rehospitalization rates
Wang L et al, 2017 [20]	Luohe, Henan Province	Quasi-randomized Trial (Patients grouped by visit date)	Outpatients with depression	CCMD-3	17-item HAM-D >17 pre-treatment; at least junior high school reading level	40 per group; 2 groups	Intervention group 16/20; Comparison group 17/23	Intervention group 33.5 (11.0); Comparison group 33.1 (10.9)	Intervention group 1 month to 10 years; Comparison group 1 month to 9 years	WeChat program on psycho-education and self-management (12 weeks).	Discharge nursing as usual	17-item HAM-D; medication adherence; GQOLI-74
Wang X et al, 2018 [21]	Qingdao, Shandong Province	Clinical trial (Allocation method not reported)	Patients with depression discharged from hospital	Not reported	Not reported	30 per group; 2 groups	Intervention group 12/18; Comparison group 14/16	Intervention group 32.6 (9.2); Comparison group 33.0 (9.7)	Intervention group 7.2 (5.7) months; Comparison group 5.8 (8.3) months	WeChat program on psycho-education, self-management, and group chats supervised by nurses (6 months)	Discharge nursing as usual	17-item HAM-D; MMAS; Nursing satisfaction
Xie H et al, 2016 [22]	Zhenjiang, Jiangsu Province	RCT	Patients with depression discharged from hospital	ICD-10	24-item HAM-D ≥21 pre-treatment	106 per group, 2 groups	Intervention group: 38/66; Comparison group 39/67	Intervention group 36.9 (8.0); Comparison group 37.1 (8.1)	Intervention group 3.9 (1.4) years; Comparison group 3.9 (1.3) years	WeChat program on psycho-education, self-management, video games, and group chats supervised by nurses (3 months)	Discharge nursing as usual	GSES; ITAQ.
Xie H et al, 2018 [23]	Zhenjiang, Jiangsu Province	Single group, pre-post design	Patients with depression discharged from hospital	ICD-10	24-item HAM-D ≤8	108, single group	57/51	38.7 (9.9)	3.2 (1.2) years	WeChat program same as Xie H et al, 2016 above (6 months)	N/A	ITAQ; BMQ; MMAS.

Quality Assessment

**Figure 2:**
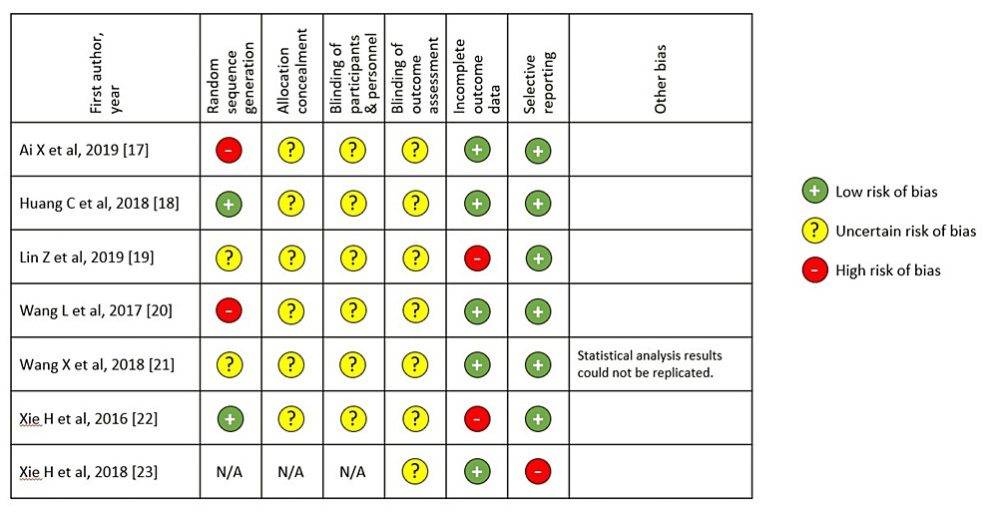
Summary of Risk of Bias for included studies

Figure 2 summarizes the results of the Risk of Bias for the included studies. All the studies had categories with high or uncertain risk of bias, notably in random sequence generation, allocation concealment, blinding of participants, and outcome assessment.

Narrative Review of Studies

Ai and colleagues conducted a quasi-randomized study on newly discharged patients with depression by ICD-10 criteria [17]. They assigned patients by order of discharge to an intervention group (n=79) that received support via an intensive WeChat-based program or to a comparison condition of usual care (n=77). They did not indicate if any blinding took place. Two components were included in the WeChat program: regular information sessions three times a week focused on psychoeducation and medication guidance, and group chats with two nurses and an unspecified number of other patients. The group chats included practical problem solving of daily challenges, medication adherence, social skills and vocational coaching, and family environment optimization. Additionally, those in the WeChat group were taught distress tolerance and mindfulness skills, encouraged to practice healthy diet and exercise habits, and instructed to avoid substances including caffeine and alcohol. Outcomes included the Self-rating Depression Scale (SDS) and Self-rating Anxiety Scale (SAS), both validated for and normed in China, and medication adherence. The SAS and SDS scores were similar between groups at the time of discharge, but by the three-month follow-up and continuing through to one year, the WeChat group had significantly lower SDS and SAS scores compared to the care as usual group (p<0.01). Both groups had comparable and high medication adherence rates at baseline, but by three months, the WeChat group outperformed the usual care group significantly, with similar rates persisting to the one-year follow-up. At one year, five of 79 (11%) were nonadherent with medications in the WeChat group compared to 35 of 77 (45%) in the comparison group (p<0.01).

Huang et al. conducted an RCT on patients with depression and anxiety who were recently discharged from the hospital. Patients were randomized to the WeChat intervention group (n=50), which was supervised by a multidisciplinary team of physicians, nurses, and psychological counselors, or to the comparison group (n=50) that received routine nursing care. The intensive intervention was available via WeChat and telephone and included psychoeducation, medication adherence guidance, family involvement and follow-up, nursing guidance, and twice-weekly supervised group chats. The SDS and SAS were used for inclusion and as outcomes. Additional outcomes included the World Health Organization Quality of Life scale (WHO-QOL) and a study-specific medication adherence scale. After six months, SDS and SAS scores in the WeChat group were significantly lower compared to the usual care group (p<0.05). The medication adherence (p<0.001) and quality of life (p<0.05) of patients were also significantly better in the WeChat group.

Lin et al. conducted an RCT with patients with depression according to the ICD-10 criteria, newly discharged, and in remission. The intervention group (n=45) received an intensive WeChat program covering psychoeducation for patients and families, media resources and activities disseminated in group chats supervised by nurses, and medication management and tracking. The comparison group (n=45) received usual care with psychoeducation. Both groups were prescribed escitalopram 20 mg daily and quetiapine 200 mg twice daily. The study is described as double-blind but it is unclear how this was achieved. Outcomes assessed were the 17-item Hamilton Depression Rating Scale (HAM-D), Hamilton Anxiety Scale (HAM-A), and the 14-item Activities of Daily Living scale (ADL). The authors reported significantly better outcomes at six months favoring the WeChat group, with large effect sizes and p<0.005 for all three clinical outcomes (HAM-D, HAM-A, ADL) compared to the comparison group. There were no significant differences between groups in relapse or rehospitalization rates during the six-month follow-up. Of note, statistical analysis was conducted on completers only (n=74), and there were more dropouts in the WeChat group than in the comparison group (10 vs 6, respectively).

Wang and colleagues conducted a quasi-randomized study in which 80 outpatients with MDD with a 17-item HAM-D score >17 were assigned into two groups according to visit order [20]. HAM-D scores at baseline were not reported. The intervention group received a WeChat program with psychoeducation (medication management, identifying and managing negative cognitions, stress management) delivered one to two times a week via text/video/graphics, and a daily chat group supervised by nurses and psychologists. The comparison group received routine health education. After 12 weeks, the WeChat intervention group had superior rates of HAM-D recovery/improvement (90% vs 68%, p<0.05) and medication adherence (88% vs 60%, p<0.01) compared to the usual treatment group. In addition, the quality of life was significantly better in the WeChat group compared to the usual treatment.

Another clinical trial involved two groups with 30 participants per group but the group allocation methods were unclear [21]. Furthermore, the diagnostic criteria, inclusion criteria, and outcome scores at baseline were not reported. The intervention group received a WeChat program with psychoeducation and self-management and a group chat for at least 0.5 hours/week, supervised by a multidisciplinary team. The comparison group received routine discharge nursing. Outcomes were assessed with the 17-item HAM-D and the Morisky Medication Adherence Scale (MMAS). After six months, the authors reported that the WeChat group had significantly lower HAM-D scores (t-test, p<0.05) than the comparison group; however, we could not replicate that result using the reported means and standard deviations (7.21 ± 3.73 versus 7.63 ± 4.12, respectively, p=0.68). The WeChat group also had significantly better medication adherence (higher MMAS scores, p<0.05) and higher overall nursing satisfaction than the comparison routine discharge nursing group.

Xie et al. conducted two studies: the first was an RCT [22] that randomized 212 patients with MDD, aged 18-55, who were discharged from the hospital and recovered, into two groups (although data on only 210 patients were reported). The intervention group (n=104) participated in an intensive program with daily activities over WeChat managed by the treatment team (psychiatrists, nurses, counselors). The WeChat program was tailored according to an individual assessment to include psychoeducation (focused on controlling symptoms and encouraging medication adherence), personalized life and social skills guidance via text messaging, video games, and a weekly group chat with nurses. The comparison group (n=106) received usual discharge care. Outcomes evaluated were the General Self-Efficacy Scale (GSAS), knowledge of depression (assessed by the Insight and Treatment Attitudes Questionnaire, ITAQ), and the MMAS. At the three-month study endpoint, the scores on all the outcome scales were significantly improved from baseline in the WeChat group, but not in the usual care group. The WeChat group also had significantly better scores at the endpoint.

The second study by Xie et al. was a single-group study [23] in patients with MDD (n=108), recently discharged and in remission (24-item HAM-D score ≤8), using a WeChat program similar to that of the first study [22]. Outcome assessments again included the ITAQ and MMAS, with scores reported at the one-month and six-month timepoints (but not at baseline). ITAQ and MMAS scores of patients at six months were significantly improved from those of the one-month time point.

Discussion

To our knowledge, this systematic review is the first to examine the strength of evidence for mHealth for depression in China. Our literature search found only seven studies of mHealth, all involving the WeChat platform. However, as assessed by the Cochrane Risk of Bias tool, the quality and reporting of studies were poor, with important information lacking and weaknesses in the study methodology. Randomization method, allocation concealment, and blinding of participants and assessors were often not reported or addressed. The study methodology was heterogeneous, with intensive, multifaceted, study-specific WeChat interventions that were difficult to compare across trials. All the studies used usual care as the comparison condition, which does not control for the intensity of professional contact and attention provided by all the WeChat interventions. Of the studies that included group chats, there is little information on the format (WeChat supports video chat, telephone calls, voice messaging, and texting) or intensity of clinician-to-patient interaction, and whether this was standardized. The outcome assessments also varied from study to study. With those caveats, the seven studies from China reported that mHealth services provided via WeChat appeared highly effective for reducing depressive symptoms, increasing medication adherence, and improving quality of life.

One potential benefit of mHealth is its scalability for delivering services to more patients in China. There are shortages in professional mental health staff, with only 2.19 psychiatrists and 5.51 registered psychiatric nurses per 100,000 people [24]. These resources are centralized in major institutions with scant outpatient coverage. In 2018, 97% of psychiatric services were provided in hospitals [25]. The studies reviewed here all delivered similar elements such as psychoeducation and self-management, supervised group chats, and contact with a multidisciplinary team and/or nursing staff. In this regard, the WeChat platform became an extension of virtual outpatient care. While Chinese patients prefer in-person medical interactions, they also have positive attitudes towards mHealth for depression treatment [11]. The advantages of these WeChat programs for patients could include greater accessibility (especially outside urban centers), more discreet delivery, lower costs, empowerment of patients and their families, and, especially important in the context of the global COVID pandemic, reduced need for in-person interactions.

However, there are also limitations and barriers to the wider use of mHealth in China and elsewhere. For example, access to the internet in rural China is still lagging. By the end of 2021, 57.6% of people in rural areas were using the internet, compared to 81.3% of people in urban areas [10]. Despite rural usage rapidly increasing from 35.4% in 2017 [10], there remains a large digital divide that compounds barriers to mental healthcare access.

There are several gaps in the available data representing areas for further research. Almost all the studies focused on recently discharged patients from hospitals or mental health centers, overrepresenting seriously ill patients from urban tertiary centers. Only one study involved acute treatment of outpatients at a hospital clinic, also based in an urban location. Data for patients in underserved regions, especially rural China, remain lacking. Workload burden is also a concern. Every study implemented WeChat platforms that required the involvement of healthcare professionals. A recent study surveying 225 Chinese mental health professionals about eHealth showed that roughly half are concerned about the increased workload, and two-thirds feel they would have inadequate time and energy [11]. Future studies will need to consider the implementation of mHealth in ways that are effective for patients and acceptable to healthcare providers. Additionally, privacy and security issues were not explicitly discussed in the included studies. Data privacy in mHealth is a major issue globally, and China has unique challenges in this regard. Some health data, such as genetic data, is already considered and regulated as property of the state [26]. China has introduced recent regulations, including the Personal Information Protection Law applying to any online data collection [27], but effective mHealth privacy regulation remains an ongoing area of development in China, as it does in many other countries [28-29]. Future mHealth studies should address privacy and security safeguards within the context of evolving regulatory changes.

Research into mHealth is challenged by the large number but low quality of apps. Even in North America, where markets are more mature and more diverse options are available, the majority of apps are not evidence-based and some are occasionally harmful [30]. In China, the total number of mental health-related apps available on online storefronts is growing rapidly, and most of them are also not evidence-based [31]. With an often-overwhelming number of options, there is a great need for future research to refine and identify the best mHealth implementations and strategies to guide patients and practitioners.

## Conclusions

In summary, compared with the expanding use of mHealth applications in other countries, mHealth in China is still in the early stages. There is preliminary positive evidence for using WeChat to improve outcomes for patients with depression discharged from the hospital. However, attention to major gaps in the evidence-base and increased rigor in study methodology are required to adequately demonstrate the efficacy, safety, acceptability, and accessibility of mHealth applications in China.
